# Modeling Joint Exposures and Health Outcomes for Cumulative Risk Assessment: The Case of Radon and Smoking

**DOI:** 10.3390/ijerph8093688

**Published:** 2011-09-13

**Authors:** Teresa Chahine, Bradley D. Schultz, Valerie G. Zartarian, Jianping Xue, SV Subramanian, Jonathan I. Levy

**Affiliations:** 1 Harvard School of Public Health, Harvard University, 677 Huntington Avenue, Boston, MA 02215, USA; E-Mails: svsubram@hsph.harvard.edu (SV.S.); jonlevy@bu.edu (J.I.L.); 2 US Environmental Protection Agency, Office of Research and Development, National Exposure Research Laboratory, Research Triangle Park, NC 27711, USA; E-Mails: schultz.brad@epa.gov (B.D.S.); zartarian.valerie@epa.gov (V.G.Z.); xue.jianping@epa.gov (J.P.X.); 3 Boston University School of Public Health, 715 Albany Street, Talbot Building, Boston, MA 02118, USA

**Keywords:** residential radon, indoor air, cumulative exposure, risk assessment, lung cancer, combined risks, health disparities, disadvantaged communities, vulnerable populations, risk-based decisions

## Abstract

Community-based cumulative risk assessment requires characterization of exposures to multiple chemical and non-chemical stressors, with consideration of how the non-chemical stressors may influence risks from chemical stressors. Residential radon provides an interesting case example, given its large attributable risk, effect modification due to smoking, and significant variability in radon concentrations and smoking patterns. In spite of this fact, no study to date has estimated geographic and sociodemographic patterns of both radon and smoking in a manner that would allow for inclusion of radon in community-based cumulative risk assessment. In this study, we apply multi-level regression models to explain variability in radon based on housing characteristics and geological variables, and construct a regression model predicting housing characteristics using U.S. Census data. Multi-level regression models of smoking based on predictors common to the housing model allow us to link the exposures. We estimate county-average lifetime lung cancer risks from radon ranging from 0.15 to 1.8 in 100, with high-risk clusters in areas and for subpopulations with high predicted radon and smoking rates. Our findings demonstrate the viability of screening-level assessment to characterize patterns of lung cancer risk from radon, with an approach that can be generalized to multiple chemical and non-chemical stressors.

## 1. Introduction

Among communities and agencies working to characterize and prioritize local environmental risks, there is an increasing call for addressing risks cumulatively by considering the influence of multiple chemical and non-chemical stressors on health outcomes [1,2]. From the perspective of environmental decision-making, this has been proposed to involve an understanding of the risks of chemical stressors in the presence of non-chemical stressors that act as effect modifiers or contribute to background processes [2,3].

One of the significant challenges that arises within cumulative risk assessment involves the need to simultaneously model exposures to chemical and non-chemical stressors in a manner that takes account of common predictors and root causes, as key demographic and structural factors are rarely formally evaluated. Cumulative risk assessment is also often hampered by a lack of epidemiological or toxicological information allowing for realistic evaluation of the influence of non-chemical stressors on health risks from chemical stressors.

Radon provides an ideal case example to explore methods for conducting cumulative risk assessments, due to the multiple factors that determine levels of radon risk. First, there is evidence that current and former smokers have a higher unit risk for lung cancer per unit of radon exposure than non-smokers, indicating that joint consideration of radon and smoking patterns would be informative in characterizing the distribution of lung cancer risk from radon [4,5]. While smoking clearly includes numerous chemicals, in the context of cumulative risk assessment, we consider it as a non-chemical stressor or “lifestyle factor” to differentiate it from those chemicals under the jurisdiction of the United States Environmental Protection Agency (EPA) [2,3]. Similar studies conducted internationally have reported added value to community risk reduction efforts from joint consideration of radon concentrations and smoking [6]. Second, the attributable risk from radon is large enough that more refined information on radon concentrations, exposures, and risk would be warranted in many decision contexts. Although different risk models and uncertainty analyses employed over the years have produced a wide range of potential risk estimates attributable to radon, all results point to radon as one of the most widespread environmental hazards requiring public health monitoring and management [4,5,7–11]. Radon is the leading cause of lung cancer deaths in non-smokers, and the second leading cause of lung cancer deaths in smokers [7]. The EPA has estimated approximately 20,000 lung cancer cases attributable to radon annually in the U.S., with an average lifetime risk of fatal lung cancer of 0.73% in the U.S. general population based on the national average concentration of 1.25 pCi/L [7]. However, having only national-scale risk estimates available can cause radon to be underappreciated in community-based risk prioritization. Radon zone maps have been developed and are readily available, but these zone maps reflect radon concentrations rather than attributable risk, and communities in an area with low or moderate radon potential may discount radon even if its risks may exceed those associated with issues of higher current visibility.

Third, both residential radon concentrations and smoking prevalence are highly variable across different locations and different populations in the U.S. Previous studies have examined each separately, but with some key limitations, and no study has jointly evaluated the demographic and geographic patterning of variables associated with radon and smoking and the subsequent patterning of radon risk.

More specifically, the distribution of residential radon concentrations across the U.S. has been the subject of numerous studies for the past several decades, and is related to both geological and housing characteristics. Radon originates from radium in underlying bedrock, the composition of which is determined by rock type and origin. Radon travels through soil and infiltrates built structures through cracks, cavities and construction joints [12]. Soil type, texture, moisture and permeability affect the movement of radon gas, in combination with climate and meteorology [13,14]. One of the primary drivers of the movement of radon from the soil into the indoor environment is pressure gradients, which can be caused by temperature differences, wind, and building heating or ventilation [15–17].

Due to the complex interplay of the factors described above and the lack of data on soil permeability to gas, it can be challenging to model radon concentrations, and previous investigations have had some limitations. The U.S. Geological Survey assigned a radon potential score by geological province based on expert evaluation of available geological and soil surveys, but could not capture local variability in soil and housing factors due to lack of local data on these factors [18]. The U.S. EPA added to the above score by incorporating measured concentrations and architecture information from state residential radon surveys to produce a national zone map of estimated radon levels by county [19]. However, state databases are of varying quality and present considerable challenges for developing nationally-consistent radon concentration estimates; in addition, they are largely based on short-term screening measurements which are limited for providing the long-term estimates needed to determine lung cancer risk. Long-term measurements are available in a limited number of state surveys, but are most well-represented nationally in the National Residential Radon Survey (NRRS) [20]. A study using measurements from the NRRS estimated median long-term residential radon concentrations by county, but cautioned that variability within a county could be significant and did not include information necessary to link with smoking data [21]. While numerous studies such as the above have reported limited predictive power in modeling radon concentrations [22–27], especially given the high level of variability and lack of sufficient local data, an approach that characterizes demographic and geographic patterns in a manner that allows for linkages with smoking models has promise for providing screening-level cumulative risk estimates.

For smoking, there are similar limitations in the prior literature in terms of comprehensively incorporating geographic and demographic variability. Smoking statistics are available at a national and state level, and local data are available in some communities but are not systematically collected and reported across the U.S. Variability in smoking has been related to compositional factors (individual demographic characteristics and socioeconomic indicators) and contextual factors (neighborhood characteristics, local and state legislation) [28–31]. These previous studies have quantified the association between smoking and compositional and contextual factors across different populations and places, but no studies have provided models with sufficient geographic and demographic stratification and coverage to allow for a refined examination of lung cancer risks from residential radon exposure in the U.S.

Despite the challenges of jointly modeling exposures to radon and smoking, community groups have repeatedly asked for assessments that take account of significant non-chemical/lifestyle stressors, and the smoking and radon interaction is one of the best understood and tractable interactions [2,32]. While no model can eliminate the need for radon measurements in each home, especially given the high risks from radon, a joint exposure assessment and model of radon-related health risks given smoking patterns can provide screening-level estimates for community groups and individuals in understanding the relative importance of radon in their communities, *versus* other issues of environmental concern. In this study, we develop a systematic approach to model both radon and smoking at high spatial and demographic resolution across the U.S., linking multiple national databases and capturing common predictors using a multilevel modeling framework [33]. We construct a multilevel regression model predicting radon concentrations using only sociodemographic and geographic covariates that can be included in a multilevel regression model predicting smoking, in order to link the two in a community-scale risk assessment of radon in the presence of smoking. We use predictors which are available across the U.S. from the Census, and leverage components of the EPA national risk assessment to develop a framework to provide communities and decision-makers with more refined estimates of lung cancer risk from residential radon exposure.

## 2. Experimental Section

### 2.1. Conceptual Framework

To capture variability in parameters affecting radon risk, we built three statistical regression models using three national datasets described below. The first model provides estimates of residential radon concentrations based on locational information and house type; the second model provides estimates of house type based on occupants’ sociodemographic characteristics, and the third model provides estimates of individual smoking status based on the same sociodemographic characteristics. Together, these regression models provide estimates for the association of sociodemographic and geographic variables with radon concentrations and smoking prevalence (Table 1). Of note, each of the three models is constructed at either the housing unit or individual level, allowing us to subsequently combine parameter estimates from the three models to produce radon exposure estimates and smoking prevalence estimates for any location (e.g., county or census tract) based on its geography and composition.

As individual-level multivariate sociodemographic and geographic data are not nationally available, we instead calculate exposures and risks for subpopulation groups at high geographic resolution, which can then be aggregated to provide patterns of population health risk. The U.S. Census provides cross-tabulated data on the number of people by age, sex, race, and poverty status, at levels of geographic resolution down to the census tract (small statistical subdivisions of a county, usually containing between 2,500–8,000 persons) [34]. Census tracts are therefore relatively small geographic entities with sufficient population size to yield cross-tabulated demographics, and were also designed to be homogeneous with respect to population characteristics, economic status, and living conditions. Within the present analysis, we present all exposure and risk calculations at county resolution for ease of presentation and proof of concept, but our individual-based analysis plan can ultimately provide smoking and radon concentration estimates at higher resolution, albeit with increased uncertainty.

### 2.2. Radon Concentration Model

We developed our radon model from the NRRS, during which long-term measurements of radon concentration were taken in all living levels of a nationally representative sample of homes from 1989–1990 [21]. While somewhat outdated with respect to current housing stock, it represents the most robust and geographically representative data set publicly available. Information on housing characteristics collected in the NRRS was combined with data from the USGS and soil surveys based on the location of each home, which was then discarded for confidentiality reasons [35]. We developed a log-linear model to quantify associations at the household level between geological and meteorological variables, housing characteristics, and annual average radon concentrations averaged over all living levels. Radon concentrations were scaled to adjust negative measurements recorded by the survey instruments to match minimal outdoor radon concentrations based on methods previously published by Price *et al.* [21]. Analysis was conducted using MLwiN 2.16 [36].

To account for the geographic clustering of samples in the NRRS we built a four-level model of housing units (n = 5,336) nested within secondary sampling units (SSU, n = 977) nested within primary sampling units (PSU, n = 125) nested within states (n = 44). Indicator variables were used to represent Census Region. In the survey primary sampling units corresponded to one or more counties, and secondary sampling units corresponded to census tracts or parts of census tracts within the sampled counties.

We selected potential covariates based on results of previous statistical analysis of the NRRS data [37]. At the county level, we included meteorological variables from a national meteorological database [38]. Alaska and Hawaii were excluded from the analysis because complete data were not available for these states within the databases most appropriate for this assessment. Geological variables (soil texture, permeability, underlying bedrock, equivalent uranium) were evaluated both separately and using a summary score of geological radon potential provided by the USGS [39]. The USGS score was provided within geological province boundaries; we assigned a score to each county based on the geological province in which the county is located, and for those counties located at the intersection of more than one geological province we assigned the score of the province which covered the largest area of the county.

At the household level, while numerous home characteristics would theoretically be linked with residential radon levels, our model structure (Table 1) necessitated that we restrict potential covariates to those available in the American Housing Survey (AHS) [40]. The AHS is the primary source of data for the U.S. housing stock and would allow for linkages with geographic and sociodemographic predictors of interest. We tested for statistically significant associations between housing variables and log radon concentrations in univariate and multivariable models, and performed chi-square tests to measure correlations between the housing variables. We then assessed the predictive power of different housing variables by comparing the reduction in variance at the state, PSU, and SSU levels in different models, as well as overall fit using log-likelihood ratio tests.

### 2.3. Housing Model

In order to apply the radon concentration model across the U.S. in a manner relevant to cumulative risk assessment, we needed to link housing characteristics predictive of residential radon with sociodemographic and geographic data available in all locations. We developed a multinomial logistic regression model to quantify associations between housing type for individual homes and publicly available sociodemographic and geographic covariates from the U.S. Census. As a result of the model-building described above, housing type was divided into five mutually exclusive categories: single detached unit with basement; single detached unit with crawl-space; single detached unit with slab-on-grade; other single detached unit; and all other units (which include attached units and mobile homes). Analysis was conducted using SAS 9.2 (SAS Institute Inc., NC, USA). Because the dependent variable has the same value for all individuals nested within a household, a multilevel model is not possible, thus the clustering of individuals within households is not accounted for in our model. State and county identifiers were not provided in the AHS dataset; metropolitan statistical area was identified for less than half of the houses and was thus not included in our analysis. Therefore Census Region was the only geographic covariate.

### 2.4. Smoking Model

We used a multilevel logistic modeling approach to develop predictors of smoking, using individual-level data from the 2006–2007 Current Population Survey-Tobacco Use Supplement (CPS-TUS). This approach has been described in detail elsewhere [41]. For the purposes of the current study, the binomial outcome modeled was ever-smoking rather than current smoking only, as the unit risk for lung cancer from radon exposure differs for non-smokers compared to ever-smokers (which includes former and current smokers). Covariates were: individual-level variables that would be available from Census cross-tabulations (age, sex, poverty, race), area poverty at the CBSA (core-based statistical area) level, and tax laws and legislation at the state level. Analysis was conducted in MLwiN 2.16.

### 2.5. Exposure and Risk Estimates

Census 2000 Summary File 3 tables were obtained to provide the number of people in each sociodemographic bin (as defined by age, sex, race, and poverty status) in each county in the U.S. Because our smoking model was based on an adult study population, we included only individuals aged 18 and above in the risk calculations.

To estimate radon risk, we first determined the predicted probability of ever-smoking for all of the individuals in each sociodemographic bin in each county by summing fixed effects of age, sex, race, poverty status, CBSA poverty, state tax, state legislation, and previous state smoking prevalence, in addition to state and CBSA residuals. Subpopulations with Black race were also assigned state-specific effect estimates for race. One hundred and eighty CBSAs were not included in the CPS sample, and only state residuals were applied for these. Second, the predicted probability of each housing type was calculated for all individuals in each bin based on the housing model by summing fixed effects of age, sex, race, poverty status, and Census Region. Third, for each housing type, the predicted radon concentration was calculated for all individuals in each bin by summing fixed effects of age, sex, race, poverty status, county meteorological variables, radon geological potential score, and state residuals (as well as county residuals for the 125 counties that were included in the NRRS sample). Five states were not included in the NRRS sample and were assigned zero residuals. Thus, we obtained estimates of the prevalence (predicted probability) of ever-smokers and of the five different housing types and corresponding radon concentrations for each sociodemographic bin in each county.

Based on this information, we estimated the population average risk associated with radon exposures for each county, following the EPA risk assessment algorithm (Table 1) and assuming (lacking evidence to the contrary) that there is no differential distribution of non-smokers and current/former smokers among the different housing types within each geographic and demographic subpopulation. We applied an exposure rate of 0.144 working level months (WLM) per year for each pCi/L of radon gas, assuming that on average people spend about 70% of their time indoors at home, and that the equilibrium fraction for radon progeny is 40% [42]. WLM is the cumulative exposure measure used in the epidemiologic literature on uranium miners, from which the unit risk factors for lung cancer were derived. The central estimates for unit risk factors per WLM are 0.00106 and 0.000851 for male and female ever-smokers; 0.000174 and 0.000161 for male and female nonsmokers, respectively [4]. Although debates have been published in the scientific literature concerning discrepancies between previous studies, there is largely a consensus on the unit risk factor established by the National Academies for indoor radon risk assessment [5,43,44], supported by recent meta-analyses which found comparable unit risk factors among the general population as in the study of uranium miners [45,46]. These meta-analyses also indicated comparable odds ratios for radon among smokers and non-smokers, which would indicate a significantly greater unit risk factor for smokers given the higher baseline risk of lung cancer, consistent with our assumptions.

We note that there are appreciable uncertainties in these radon risk calculations, given uncertainty in the regression models for radon concentrations, home type, and smoking prevalence; uncertainty in the unit risk factors for radon and the extent to which effect modification by smoking occurs; and broad-based uncertainty related to the representativeness of the NRRS measurements, assumptions of a stationary population, and so forth. A comprehensive Monte Carlo analysis to characterize the magnitude of the uncertainties was not conducted because it was not considered informative in light of the complex multivariate structure of the regression models and the number of important factors that would elude quantification. Instead, following recent guidance to conduct uncertainty analyses that relate to the decision context and increase understanding about the problem under study [1], we provide some quantitative and qualitative uncertainty information to determine whether our core conclusions are robust.

## 3. Results and Discussion

### 3.1. Radon Concentration Model

At the county level, the USGS summary score had higher statistical significance and improved the fit of the model more than the separate geological and soil covariates (as assessed using log-likelihood ratio tests). Annual heating infiltration degree days and average diurnal temperature difference were retained as meteorological variables (Table 2).

At the house level, statistically significant variables which improved the fit of the model and were available in the AHS dataset were: type of unit (detached *vs.* attached), presence of basement, presence of central air conditioning, use of gas fuel for heating, use of steam or hot water distribution system for heating, number of gas appliances, and year built. However, chi-square tests showed multiple correlations between these housing variables, and the use of numerous housing variables complicates linkages with individual Census data. We fit a model containing a five-category house type variable (type of unit, basement) and it explained 85% of between-state variance, 51% of between-county variance, and 25% of between-census tract variance, compared to a model including all housing variables which explained 86% of between-state variance, 50% of between-county variance, and 29% of between-census tract variance. We therefore utilized the five-category house type variable in subsequent analyses. The reference groups for categorical variables in this model were detached homes with basements; Midwest geographic region; and Low Geological Potential.

Census Region was a statistically significant predictor (p = 0.005), although with no statistically significant differences among the South, West, and Midwest. All county-level and house-level covariates in the final model were significant, with the exception of the crawl-space indicator in the house type variable.

### 3.2. Housing Model

House types were significantly associated with Census Region, poverty status, age, and race. (No significant differences were observed by gender.) White subpopulations living above the poverty threshold in the Midwest had the highest odds of living in detached homes with basements compared to attached homes. Subpopulations with the lowest odds of living in detached homes with basements compared to attached homes were Black race, below poverty threshold, ages 25–34, living in the West. Parameter estimates for the housing model are presented in Appendix I.

### 3.3. Smoking Model

The prevalence of ever-smokers in the CPS-TUS 06–07 was 38.6% (17.9% current smokers, 20.7% former smokers). Associations of sociodemographic variables with ever-smoking were comparable to the associations reported previously by the CDC using CPS-TUS data for current smoking prevalence, with a few exceptions [47]. The inverted U-shaped association for age peaked at a higher age than in the model for current smoking prevalence. State legislation restricting smoking in public venues and percent poverty at the CBSA level were not significant predictors of ever-smoking and did not show the same directionality as for the previously published current-smoking model. State cigarette excise tax showed a significant negative association with ever-smoking; this association persisted after controlling for previous state smoking prevalence, and is therefore not likely due to endogeneity or reverse causation. Men showed higher odds of smoking than women, and this effect was modified by race. The variance of the random parameters at the state and CBSA levels were 0.005 and 0.040 respectively, compared to 0.004 and 0.013 in the previously published current smoking model, which was not constrained to covariates available for all subpopulations nationally. Parameter estimates for the smoking model are listed in Appendix II.

### 3.4. Exposure and Risk Estimates

**Figure 1:**
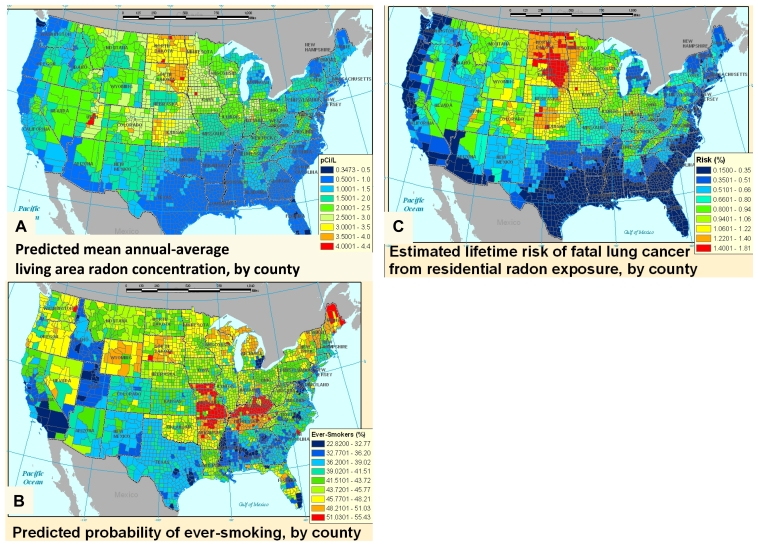
(**a**) Spatial patterns of county-average radon concentration, (**b**) Smoking, and (**c**) Lung cancer risk associated with radon.* * Note that county-average risks include significant heterogeneity, and this figure cannot be used to identify individual homes as not needing to measure radon concentrations.

Central estimates for county average lifetime radon lung cancer risk estimates ranged from 0.15% to 1.8%, with a mean by county of 0.66% and a median of 0.64% (standard deviation = 0.3%), in agreement with the national average risk of 0.7% previously reported by the EPA [7]. High-risk clusters were observed in the northern Midwest states, which had relatively high predicted levels of both radon and ever-smoking; South Dakota in particular shows a number of counties which contained among the highest estimated mean radon concentrations (Figure 1a), and the same counties were also on the higher end of estimated ever-smoking prevalence (Figure 1b). Two of the six counties nationwide which show predicted mean concentrations greater than 4 pCi/L were observed in Utah; however, because Utah has among the lowest smoking rates in the country, these counties did not emerge among the highest risk counties in the risk map (Figure 1c). High-smoking clusters were predicted in selected states in the Midwest and Southeast, where radon concentrations were on the lower end, and therefore risk clusters did not emerge in these states. Missouri and Kentucky in particular were among the states with the highest predicted probability of ever-smokers, but had average radon risk levels. Coastal states had the lowest radon concentrations, and many of these were also below-average smoking states, therefore resulting in the lowest average population risk. The population-weighted average risk for the continental U.S. was lower than the above-mentioned mean county-level average of 0.66%, at 0.5% (Appendix III), given low risk clusters in some heavily populated areas. Population-weighted national average values for radon concentration and ever-smoking prevalence were in agreement with previous results published by the EPA and CPS-TUS at 1.3 pCi/L and 38.6% respectively [7,47]. The lower national risk in our study relative to the EPA risk assessment may be attributable in part to correlations between radon and smoking in our dataset.

### 3.5. Patterns of Variability

The interplay between radon concentrations and smoking, and the demographically and geographically variable nature of both, results in a spatial distribution of radon-related lung cancer risk within a cumulative risk assessment framework that has not been captured in previous studies. Although there is considerable uncertainty in the estimates (see Section 3.6 below), the models represent previously-documented spatial patterns of each of the individual exposures, and help illustrate the influence of including smoking patterns on the estimated spatial variability of radon-related risk.

Comparing the radon risk map and concentration map shown in Figure 1, the patterns follow a similar trend in many places but are far from identical. The shifting of patterns between the two maps can be illustrated by comparing areas with similar radon concentrations but different smoking patterns; for example, while Indiana does not stand out as a high radon area in the concentration map relative to Utah, its risk levels are higher due to the large difference in smoking prevalence between the two states. Comparing our risk map with previous screening maps such as the EPA map of radon zones, the overall patterns agree but nuances emerge within the highest potential zone, as illustrated by the high risk cluster in the northern Midwest. Whether this is a reflection of uncertainty in our models or the use of categorical rather than continuous outcomes in the EPA radon zone map would require further exploration.

The underlying Census sociodemographic data behind our risk map form a key factor which contributes directly to the smoking predictions through the strong association between compositional variables and smoking prevalence, and indirectly to the radon concentration predictions through the housing model component. For example, white men in the Midwest region have higher odds of living in detached single units with basements and have higher odds of smoking. When such individuals are located in counties with high geological radon potential and higher than average diurnal temperature swings and total infiltration heating degree days, their lung cancer risk from residential radon exposure will likely exceed the national average. On the other hand, multi-directionality in exposure and risk factors was also observed; for example, living below the poverty threshold was negatively associated with the presence of a basement, thus likely to have lower radon concentrations after controlling for location, while it was positively associated with probability of smoking. Cumulative risk assessments that capture the positive and negative correlations among chemical and non-chemical stressor exposures will provide a more nuanced characterization of individual and subpopulation health risks. Broadly, jointly examining the patterns of demographic and geographic predictors associated with radon and smoking allows for identifying the locations of clusters with the highest predicted probability of lung cancer from residential radon exposure given effect modification associated with smoking.

### 3.6. Uncertainty Characterization

As described above, given our application of multiple multivariate regression models and the presence of numerous uncertainties that cannot be readily quantified, formal propagation of uncertainty does not provide readily interpretable information. Given the context of our work, we are primarily concerned with whether any of the assumptions in our analysis could invalidate either the approach or the general spatial patterns presented in Figure 1. With this perspective in mind, we evaluate key uncertainties both quantitatively and qualitatively below.

One of the key assumptions in our analysis is the unit risk factor for radon and the evidence for effect modification from smoking. In its assessment of risks from radon in the home, EPA conducted a Monte Carlo simulation to characterize uncertainty in the unit risk factor for lung cancer from radon, though with the caveat that many factors were omitted [7]. The resulting estimates for risk per WLM had a median estimate of 0.00098 with a 90% uncertainty interval of 0.0004–0.0020 for ever-smokers; and a median estimate of 0.00054 for the general population (including ever-smokers and never-smokers) with a 90% uncertainty interval of 0.0002–0.0012. This suggests that the unit risk factors are accurate within a factor of 2–3, albeit with many significant uncertainties omitted. If applied across the board, this would influence the magnitude of our risk estimates but not the patterns.

A more significant question from the perspective of our analysis is whether effect modification due to smoking is robust, as the omission of this factor would imply that the patterns of radon-related lung cancer risk would closely resemble concentration patterns (and obviate the need to account for smoking patterns). While this is clearly uncertain, we note that the effect modification is submultiplicative, with a higher odds ratio for never-smokers than ever-smokers. The large difference in risk per WLM is largely attributable to the much higher baseline risk of lung cancer among smokers, an assumption with little uncertainty. Thus, unless the odds ratio of lung cancer from radon were an order of magnitude lower for ever-smokers than never-smokers, which seems unlikely, the risk per WLM would remain higher for ever-smokers and our general conclusions about the importance of accounting for smoking would remain robust [4,48,49].

Going beyond risk per WLM, while EPA reported that the uncertainty in the exposure parameters (radon concentration, decay rate, time spent at home) are minor compared to uncertainty in the unit risk factor [7], in our study the reverse is likely to be the case. Because we rely on three linked regression models to capture variability in radon exposure and smoking prevalence, it is not only the magnitude of risk that is subject to considerable uncertainties, but also the patterns of risk variability which our models capture. However, quantifying uncertainty across these models would be technically challenging, given the need for information on the covariance among the parameter estimates and across the regression models, and would omit many key factors. We instead discuss important uncertainties that go beyond the standard errors reported in our three regression models.

For example, while the sociodemographic data employed in our model captures variability in housing types and smoking patterns with respect to the national average, many factors remain which influence these outcomes and which were not included as predictors in our models, given a lack of available cross-tabulated data. These include other socioeconomic indicators (for example education, occupation, immigrant status, marital status) as well as contextual factors (for example local variations in construction patterns and smoking restrictions). Additionally, it is important to note that our risk model implicitly assumes that lifetime radon exposures are correlated with predictions based on the current residential location. The migration of individuals and populations from one part of the country to another would clearly complicate radon exposure models and would tend to blur the association between current location and risk.

Our models have a number of additional uncertainties and should be considered for illustrative or screening purposes only. As in any statistical regression, statistical inferences are drawn about the true population distribution based on a limited number of samples. The NRRS is nationally representative but included samples from only 125 counties and does not capture housing structures built within the past 20 years. Although the radon concentration model benefited from a multilevel structure in which state and county effects were drawn from a random distribution, random parameter variance was not fully captured by the available data. Thus there remain unexplained state and county effects, and some high radon concentration counties were not captured, such as those in eastern Pennsylvania and northwestern New Jersey. Another limitation is that the housing model did not benefit from a multilevel structure because of the nature of the outcome variable, which is the same for all individuals in a household. Among the predictors of the housing model, poverty status is the most relevant for interpretation because it is a shared household characteristic (although measured as individual level variables in this dataset). On the other hand, sex and age and race are individual characteristics, and the nesting of individuals within households was not accounted for due to the lack of multilevel structure. A further weakness of the housing model was lack of higher levels such as county, metropolitan statistical area, or state, which were not included due to limited geographic identifiers in the AHS. Finally, our models were limited by the lack of contemporaneous data across models; the NRRS measurements were collected in 1989–1990, and the unit risk coefficient used in our risk estimates was developed by EPA based on 1990 mortality rates and smoking prevalence, but we aimed to develop a predictive model keyed on demographic data from 2000 and smoking patterns from 2006–2007. This contributes some uncertainty to our risk estimates, though the broad-based demographic and geographic patterns of smokers and housing characteristics are relatively stable over time.

While these uncertainties are significant, the general spatial patterns of radon concentrations and prevalence of ever-smokers in Figure 1 are consistent with previous publications, indicating that we have reasonably captured the geographic areas with high/low radon and high/low ever-smoking prevalence. Similarly, the demographic and structural predictors are in agreement with previous models. Thus, the quantitative uncertainties are large for a given subpopulation within a given location, but our general conclusions about exposure variability and the importance of accounting for both radon and smoking are robust.

### 3.7. Lessons for Cumulative Risk Assessment

In spite of these limitations and appreciable uncertainties, our approach toward cumulative risk assessment offers some insights and lessons for future studies. First, in spite of the significant constraints in covariates available for our exposure models, given the need to rely on broadly available information common to radon and smoking models, we demonstrated predictive power similar to prior studies. Although radon is difficult to predict due to the local variation in soil factors for which no national-scale data are available, and our regression models used a more limited set of covariates than in previous investigations, our model was built on the only known national database of measured long-term radon concentrations in the U.S. and our radon concentration predictions compare well with previous estimates employing both long-term and short-term national and state datasets [21]. Furthermore, the multinomial house type variable retained in our final model performed well when compared to multiple individual and often highly correlated housing variables. The covariates in our ever-smoking model explained a comparable amount of state and CBSA variance to previously published smoking models, despite being constrained to publicly available covariates only.

Because of this reasonable model performance, we were able to jointly estimate the geographic and sociodemographic patterns of both radon and smoking in a manner that allows for modeling of radon risk given the influence of a key non-chemical/lifestyle stressor, relying exclusively on publicly available data. In theory, one could use our models to also estimate the direct effects of smoking on lung cancer, allowing for a comparison between these stressors. While doing so could inform certain policy applications, it would go against the general principle that cumulative risk assessment should focus on decision-relevant analyses considering plausible alternative policies [1], which would typically not involve agencies comparing radon mitigation and smoking cessation programs. Regardless, our work provides a template for a variety of applications relevant to different stakeholders and decision-makers.

This type of modeling could provide insight to communities seeking screening-level information on their radon exposure and risk, though further development and evaluation would clearly be warranted. In particular, although we presented county average estimates within this paper for ease of presentation, our focus on individual-level exposure prediction means that smaller geographic aggregates (such as census tracts) could be characterized in a computationally identical fashion. Though uncertainty would be increased for some steps in the analysis, there could be some improvements in the interpretability of our models (including for housing, where urban/rural status could be incorporated as a covariate). Future research can also improve the strength of these models by considering variability in other parameters affecting exposure and risk, such as time spent at home (which may also vary by age, sex, poverty, and race) and equilibrium fraction (which depends on factors such as particle size distributions in homes, in turn affected by smoking patterns). Model evaluation is also needed using measured long-term residential radon concentrations within selected geographic areas and demographic subpopulations, or through evaluation of lung cancer risk patterns among non-smokers. These and other local-scale validation activities could provide more meaningful insight about the magnitude of uncertainty in radon-related lung cancer risks than would be available strictly from propagation of parametric uncertainties.

Beyond the specific application to radon, our approach provides a first step in developing approaches for cumulative risk assessment at the community level. Other chemical and non-chemical stressors could be similarly evaluated, provided that there were large nationally representative data sets available (*i.e.*, the National Health and Nutrition Examination Survey). A near-term effort could involve the incorporation of additional indoor air hazards within the cumulative lung cancer inhalation risk pathway, which would leverage the present work and provide a more comprehensive cumulative evaluation. To facilitate these and other cumulative risk assessments at high geographic resolution, we recommend that national survey bureaus make efforts to provide increased geographic identifiers in public use data files such as the AHS, subject to confidentiality constraints, given the importance of location in determining variability in population exposure and risks.

## 4. Conclusions

Our models provide an approach for leveraging publicly available information from nationally representative data to capture correlations among parameters affecting both residential radon exposure and subsequent risk of lung cancer given smoking patterns. These methods provide a basis for building new frameworks for modeling cumulative risk at the community level. Similar statistical models have been developed to predict radon concentrations for individual use, but require data inputs on individual homes that would not be broadly available, and therefore cannot provide national-scale population risk estimates [50]. Our model yields similar national-average radon lung cancer risk estimates as prior risk assessments but illustrates an order of magnitude variation in average risk at county resolution, though even the lowest risk counties exhibit average lung cancer risk from residential radon exceeding one in a thousand. This research is the first to quantify variability in lung cancer risk from residential radon exposure, moving beyond the national average estimates provided in the EPA national risk assessment and explicitly capturing the influence of a key non-chemical/lifestyle stressor. While these results cannot replace individual home measurements, which are recommended by the EPA for almost all homes [51], our study can provide a key input to community-scale risk prioritization efforts within the context of cumulative risk.

## Figures and Tables

**Table 1:**
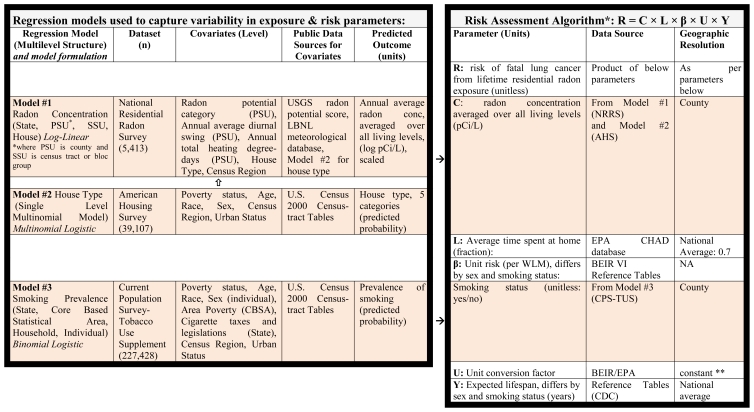
Regression models used to capture variability in parameters of risk assessment algorithm.

* Source: USEPA (2003) EPA assessment of risk from radon in homes. (EPA 402-R-03-003) [7].

** Conversion factor = [0.004 WL/(pCi/L)] × [51.6WLM/WL–y].

**Table 2:** Log-linear radon concentration model, fixed effects.

PARAMETER	ESTIMATE	SE	P-VALUE
Intercept	−.02	0.09	
Northeast	−0.35	0.12	0.003
South	0.026	0.14	0.85
West	−0.18	0.15	0.23
Medium Geological Potential	0.43	0.074	< 0.001
High Geological Potential	0.74	0.11	< 0.001
Heating Infiltration Degree-Days	0.00006	0.00002	0.008
Average Diurnal Swing	0.041	0.01	< 0.001
Attached Unit	−0.71	0.03	< 0.001
Detached with Crawl Space	−0.059	0.04	0.18
Detached with Concrete Slab	−0.4283	0.03144	< 0.001
Other Detached	−0.5308	0.1243	< 0.001

**Appendix I:** Odds ratios from Multinomial Logistic Regression of house types, AHS 2007 (Reference group = single detached houses with basements).

Covariate	Outcome	OR	95%CI	
**Poverty*vs.*nopov**	**attached unit**	4.40	4.14	4.66
	**crawl space**	1.74	1.61	1.88
	**slab**	1.40	1.30	1.51
	**other**	2.36	1.91	2.91

**Male*vs.*Female**	**attached unit**	0.97	0.94	1.01
	**crawl space**	1.01	0.96	1.05
	**slab**	1.00	0.96	1.04
	**other**	1.04	0.90	1.19

**Race: black*vs*. white**	**attached unit**	2.75	2.60	2.92
	**crawl space**	1.46	1.35	1.57
	**slab**	1.56	1.46	1.68
	**other**	1.73	1.40	2.15

**Race: asian*vs.*white**	**attached unit**	2.34	2.13	2.56
	**crawl space**	0.84	0.73	0.97
	**slab**	2.07	1.86	2.30
	**other**	2.10	1.48	2.97

**Race: other*vs.*white**	**attached unit**	1.76	1.53	2.02
	**crawl space**	1.22	1.02	1.45
	**slab**	1.50	1.28	1.76
	**other**	2.10	1.32	3.33

**Race: native*vs.*white**	**attached unit**	1.91	1.59	2.31
	**crawl space**	1.42	1.14	1.77
	**slab**	1.74	1.42	2.14
	**other**	1.83	0.96	3.50

**Region: NE*vs.*S**	**attached unit**	0.44	0.42	0.46
	**crawl space**	0.04	0.03	0.04
	**slab**	0.03	0.03	0.03
	**other**	0.09	0.07	0.12

**Region: MW*vs.*S**	**attached unit**	0.21	0.20	0.22
	**crawl space**	0.10	0.09	0.10
	**slab**	0.04	0.03	0.04
	**other**	0.12	0.10	0.15

**Region: W*vs.*S**	**attached unit**	1.35	1.28	1.43
	**crawl space**	1.04	0.98	1.10
	**slab**	1.09	1.03	1.15
	**other**	0.64	0.53	0.78

**Age: <18*vs.*45–54**	**attached unit**	1.11	1.05	1.18
	**crawl space**	0.87	0.81	0.93
	**slab**	1.07	1.00	1.14
	**other**	0.71	0.57	0.89

**Age: 18–24*vs.*45–54**	**attached unit**	1.99	1.85	2.15
	**crawl space**	1.01	0.92	1.10
	**slab**	1.09	1.00	1.19
	**other**	0.91	0.68	1.24

**Age: 25–34*vs.*45–54**	**attached unit**	2.68	2.50	2.87
	**crawl space**	1.17	1.08	1.28
	**slab**	1.31	1.21	1.42
	**other**	0.95	0.71	1.26

**Age: 35–44*vs.*45–54**	**attached unit**	1.25	1.17	1.33
	**crawl space**	0.94	0.87	1.01
	**slab**	1.08	1.00	1.16
	**other**	0.64	0.49	0.84

**Age: 55–64*vs.*45–54**	**attached unit**	1.08	1.01	1.16
	**crawl space**	1.08	1.00	1.17
	**slab**	1.04	0.96	1.13
	**other**	1.26	0.99	1.61

**Age: 65–74*vs.*45–54**	**attached unit**	1.16	1.06	1.25
	**crawl space**	1.19	1.08	1.30
	**slab**	1.10	1.01	1.21
	**other**	1.33	1.01	1.76

**Age: 75plus*vs.*45–54**	**attached unit**	1.80	1.66	1.96
	**crawl space**	1.39	1.26	1.54
	**slab**	1.10	1.00	1.22
	**other**	1.71	1.28	2.28

***Model Intercepts:***	***Estimate (SE)***			
	***Attached unit***	1.14	(0.03)	
	***Crawl Space***	−0.51	(0.04)	
	***Slab***	−0.30	(0.04)	
	***Other***	−2.80	(0.10)	

**Appendix II:** Multilevel Logistic Model of ever-smoking in U.S. adults (CPS-TUS 2006–2007).

Random Parameters	ESTIMATE	SE			

Variance of state random effect	0.005	0.003			
Variance of random slopes by state for black race	0.035	0.012			
Covariance of random effect for state and random slope for black race	−0.008	0.004			
Variance of CBSA random effect	0.040	0.005			
Variance of household random effect	0.521	0.012			

**Fixed Parameters**	ESTIMATE	SE	OR	95% CI	

*Intercept*	−*0.43*	*0.04*			
Male	0.41	0.01	1.51	1.48	1.54
Age 18–24 years	−0.86	0.02	0.42	0.41	0.44
Age 25–34 years	−0.34	0.02	0.71	0.69	0.73
Age 35–44 years	−0.28	0.01	0.76	0.73	0.78
Age 55–64 years	0.24	0.02	1.28	1.24	1.32
Age 65–74 years	0.33	0.02	1.39	1.34	1.44
Age 75 plus	−0.03	0.02	0.97	0.94	1.01
Poverty	0.37	0.02	1.45	1.41	1.50
Income not reported	−0.24	0.01	0.79	0.77	0.81
Black race	−0.50	0.04	0.61	0.56	0.66
American Indian or Native Alaskan	0.29	0.06	1.33	1.17	1.51
Asian	−1.38	0.05	0.25	0.23	0.28
Native Hawaiian or Pacific Islander	−0.34	0.12	0.71	0.56	0.90
Other/Two or more races	0.20	0.05	1.23	1.11	1.35
Black*Male	0.18	0.03	1.19	1.12	1.28
Native*Male	−0.07	0.09	0.93	0.78	1.11
Asian*Male	0.91	0.06	2.48	2.22	2.78
Islander*Male	0.07	0.17	1.07	0.77	1.48
Other*Male	0.03	0.07	1.03	0.90	1.18
State cigarette excise tax	0.08	0.03	1.08	1.03	1.14
Previous state prevalence (2003)	0.03	0.01	1.03	1.02	1.04
Indoor smoking restrictions in >6 of 7 venue types	0.01	0.03	1.01	0.95	1.08
CBSA % poverty above median	−0.02	0.03	0.98	0.92	1.04
CBSA unidentified/nonmetropolitan	0.04	0.04	1.04	0.97	1.12

**Appendix III:** Population-weighted average radon concentration, smoking, and risk by state in the continental U.S.*.

State	Concentration^1^ (pCi/L)	Ever-Smoking Prevalence^2^ (%)	Fatal Lung Cancer Risk from Radon^3^ (%)
Alabama	1.32	38.6	0.505
Arizona	0.95	38.9	0.353
Arkansas	1.00	45.9	0.426
California	1.04	31.4	0.341
Colorado	2.61	38.2	0.978
Connecticut	1.20	39.8	0.465
Delaware	0.90	41.2	0.357
D.C.	1.35	37.4	0.536
Florida	0.55	37.1	0.214
Georgia	1.12	34.5	0.385
Idaho	1.83	34.8	0.620
Illinois	1.78	39.7	0.683
Indiana	2.50	42.7	0.987
Iowa	2.56	43.4	1.041
Kansas	2.44	41.4	0.940
Kentucky	1.77	47.9	0.775
Louisiana	0.72	38.6	0.266
Maine	1.27	47.9	0.564
Maryland	1.39	32.4	0.469
Massachusetts	1.05	38.9	0.405
Michigan	1.37	37.2	0.504
Minnesota	2.64	43.1	1.054
Mississippi	0.87	35.3	0.302
Missouri	1.69	44.9	0.701
Montana	2.13	43.1	0.868
Nebraska	2.85	43.8	1.161
Nevada	1.12	37.8	0.424
New Hampshire	1.28	41.4	0.507
New Jersey	0.71	36.8	0.259
New Mexico	1.61	39.8	0.601
New York	1.04	41.5	0.422
North Carolina	1.05	39.0	0.403
North Dakota	3.39	42.1	1.350
Ohio	1.83	43.0	0.738
Oklahoma	1.00	43.1	0.404
Oregon	0.87	40.2	0.342
Pennsylvania	1.30	42.9	0.538
Rhode Island	1.10	45.2	0.472
South Carolina	1.02	39.9	0.397
South Dakota	3.16	45.2	1.294
Tennessee	1.77	43.1	0.733
Texas	0.91	35.3	0.313
Utah	2.13	27.6	0.596
Vermont	1.11	47.3	0.483
Virginia	1.51	36.5	0.557
Washington	0.83	41.8	0.331
West Virginia	1.46	43.2	0.621
Wisconsin	2.02	43.6	0.829
Wyoming	2.01	45.7	0.845

Population-Weighted National Average	1.30	38.6	0.497

1 Modeled mean annual-average living area radon concentration.

2 Modeled prevalence (predicted probability) of ever smoking.

3 Modeled lifetime risk of fatal lung cancer from residential radon exposure.

* Note that average risks include significant heterogeneity, and that each state has significant numbers of people greatly impacted by radon risk. Also, note that there is significant uncertainty in between-state comparisons, and this table cannot be used to identify individual homes as not needing to measure radon concentrations.

## List of Abbreviations

AHS: American Housing Survey
BEIR VI: Biological Effects of Ionizing Radiation VIth Committee Report
CBSA: Core-based statistical area
CHAD: Consolidated Human Activity Database
CDC: Centers for Disease Control and Prevention
CPS-TUS: Current Population Survey-Tobacco Use Supplement
EPA: Environmental Protection Agency
NRRS: National Residential Radon Survey
PSU: Primary Sampling Unit
SSU: Secondary Sampling Unit
USGS: United States Geological Survey
WLM: Working-level months
